# A large and diverse brain organoid dataset of 1,400 cross-laboratory images of 64 trackable brain organoids

**DOI:** 10.1038/s41597-024-03330-z

**Published:** 2024-05-20

**Authors:** Julian Schröter, Luca Deininger, Blaz Lupse, Petra Richter, Steffen Syrbe, Ralf Mikut, Sabine Jung-Klawitter

**Affiliations:** 1 Division of Pediatric Epileptology, Center for Pediatrics and Adolescent Medicine, University Hospital Heidelberg, Heidelberg, Germany; 2 Division of Pediatric Neurology and Metabolic Medicine, Department I, Center for Pediatric and Adolescent Medicine, Medical Faculty Heidelberg, University Hospital Heidelberg, Heidelberg, Germany; 3 Group for Automated Image and Data Analysis, Institute for Automation and Applied Informatics, Karlsruhe Institute of Technology, Eggenstein-Leopoldshafen, Germany; 4 MSH Medical School Hamburg, University of Applied Sciences and Medical University, Hamburg, Germany

**Keywords:** Disease model, Machine learning

## Abstract

Brain organoids represent a useful tool for modeling of neurodevelopmental disorders and can recapitulate brain volume alterations such as microcephaly. To monitor organoid growth, brightfield microscopy images are frequently used and evaluated manually which is time-consuming and prone to observer-bias. Recent software applications for organoid evaluation address this issue using classical or AI-based methods. These pipelines have distinct strengths and weaknesses that are not evident to external observers. We provide a dataset of more than 1,400 images of 64 trackable brain organoids from four clones differentiated from healthy and diseased patients. This dataset is especially powerful to test and compare organoid analysis pipelines because of (1) trackable organoids (2) frequent imaging during development (3) clone diversity (4) distinct clone development (5) cross sample imaging by two different labs (6) common imaging distractors, and (6) pixel-level ground truth organoid annotations. Therefore, this dataset allows to perform differentiated analyses to delineate strengths, weaknesses, and generalizability of automated organoid analysis pipelines as well as analysis of clone diversity and similarity.

## Background & Summary

### Brain organoids as cellular model of neurodevelopmental disorders

Brain organoids represent a useful research tool for modeling of pathologies of the central nervous system, especially malformations of cortical development (MCDs)^1,2^. MCDs are genetic disorders affecting one or several processes during human corticogenesis. Besides cortical and extra-cortical malformations, these conditions are frequently accompanied by alterations of the brain volume which is commonly reflected by a decreased head size, known as microcephaly^3^. In order to recapitulate microcephaly and detect potential growth alterations during early brain development, monitoring of the growth and morphology of brain organoids during cultivation is essential^4^.

### Manual versus automated organoid growth monitoring

Since volumetric measurements of brain organoids are feasible but come with an increased experimental effort^5^, brightfield microscopy is the common standard to determine organoid size and growth. Despite the widespread use of brain organoid models in neurobiological research, organoid size in brightfield images is still frequently determined manually using common imaging software^4,6^. This manual quantification is time-consuming for large sample sizes and susceptible to observer bias. To address this problem, classical image processing tools such as CellProfiler and OrganoSeg have been developed for automated organoid quantification using 2D image segmentation^7,8^. Furthermore, deep-learning-based methods such as the recently published tool MOrgAna were presented for organoid segmentation, quantification, and visualization of morphological information^9^. Other deep-learning-based methods have been developed for single-organoid detection, tracking and analysis but have primarily been validated on cancer organoid datasets or do not exist as standalone tools with a user interface for broad usage in research^10–13^.

### Our contribution

We recorded a large dataset comprising more than 1,400 images of 64 trackable brain organoids from four different clones imaged at 10 time points over 30 days and in two independent labs. To allow analyses of organoid size, growth and diversity, we generated pixel-level organoid annotations. Due to high clone diversity and distinct development, cross-laboratory images, frequent imaging, and occurrence of common imaging distractors including light reflexes due to rims of plate wells or shadows and different colors caused by culture medium, this dataset allows to perform differentiated analysis of automated organoid analysis pipelines to uncover their strengths and weaknesses. Specifically, we show with respect to two classical organoid analysis pipelines CellProfiler and OrganoSeg, and two deep-learning-based methods MOrgAna and SegFormer^14^, how our dataset delineates method generalizability to different organoid states, imaging labs and their strengths and weaknesses in certain scenarios such as the presence of common imaging distractors. Furthermore, we show how the dataset allows to investigate clone diversity and similarity.

## Methods

### ***In vitro*** methods

#### iPSC generation and culture

Induced pluripotent stem cell (iPSC) lines were generated from peripheral blood mononuclear cells (PBMCs) of a healthy control (wt2D), two patients with *TUBA1A*- and *TUBB2A*-associated tubulinopathy as well as one patient with the neurotransmitter disorder tyrosine hydroxylase (TH) deficiency^15,16^. iPSCs were cultivated in StemFlex medium (ThermoFisher Scientific) under standard conditions (37 °C / 5% CO_2_) using Matrigel-coated 6-well plates (Corning, Greiner Bio-One). Cells were propagated as clumps every 3-4 days with ReLeSR (StemCell Technologies).

#### Organoid generation and cultivation

**Fig. 1:**
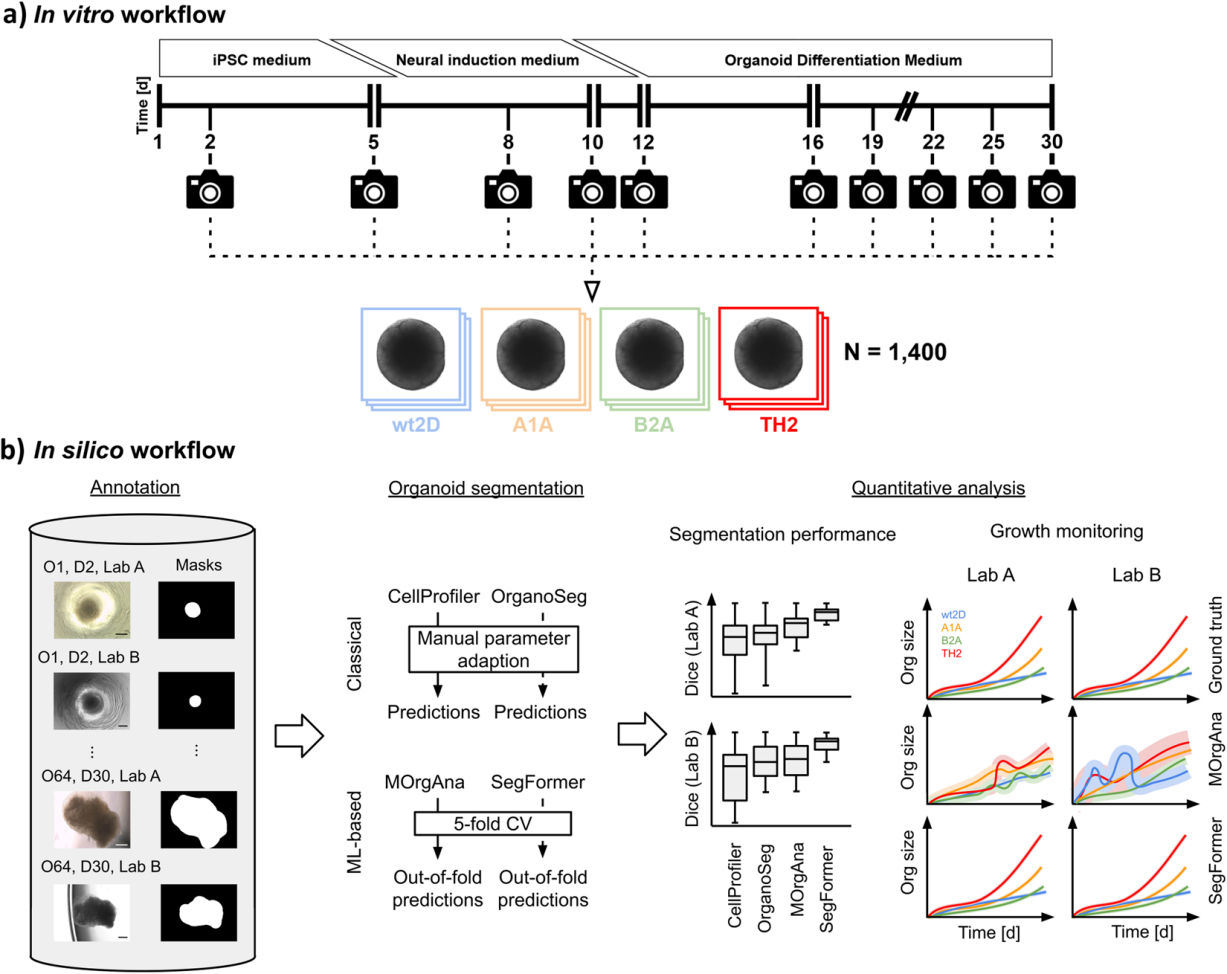
Data generation and differentiated analysis of organoid analysis pipelines. (**a**) Replicates of the four clones wt2D, A1A, B2A, TH2 were cultivated and imaged over 30 days resulting in a dataset of more than 1,400 images. (**b**) Based on this dataset, the strengths and weaknesses of methods for organoid growth monitoring can be clearly delineated. Scale bar: 500 μm.

Forebrain organoids were generated as previously described, with slight modifications^4,17^. Briefly, iPSCs were dissociated using StemPro Accutase (ThermoFisher Scientific) at 70–90% confluency on day 1. Cell aggregates were formed using 96-well V-bottom plates (Greiner) with 6,000 cells/well in 150 µl iPSC medium with 50 µM Y-27632 (StemCell Technologies). From each clone, 16 technical replicates were generated. Medium was subsequently changed daily for 4 days using iPSC medium without Y-27632. At day 5, the medium was changed to neural induction medium containing Neurobasal and DMEM/F-12 medium in a 1:1 ratio with B27 (1:100) and N2 (1:200) supplement, 1% GlutaMax, 0.5% NEAA (ThermoFisher Scientific), and 10 µg/mL heparin as well as the compounds LDN-193189 (180 nM), A83-01 (500 nM), and IWR-1 (10 µg/mL, all Tocris). Neural induction medium was changed on day 8. On day 10, LDN-193189, A83-01, and IWR-1 were removed and the resulting organoid differentiation medium was used for the remaining protocol. On day 12, organoids were embedded in Matrigel (Corning) on 10 cm petri dishes (Sarstedt) and excised from Matrigel on day 16. To allow growth monitoring of individual organoids, organoids were individually marked on the petri dishes and separately transferred into 24-well ultra-low attachment plates (Corning) after excision. Organoids were subsequently kept in agitated culture using an orbital shaker until day 30. Organoid differentiation medium was changed every 3-4 days. For a schematic overview on the culture conditions please see Fig. 1a.

#### Organoid imaging

To ensure broad applicability for large organoid batch sizes, 2D brightfield images were taken instead of 3D stacks using a confocal microscope. To reduce individual technical effects and observer bias on data analysis, forebrain organoids were separately and simultaneously imaged on two bright-field microscopes with different manufacturers and software (microscope 1: Leica DMi 1, camera 1: Leica MC170 HD, software 1: Leica Application Suite software, LAS EZ, v.3.4.0; microscope 2: Zeiss Axio Vert.A1, camera 2: Zeiss AxioCam MRc, software 2: ZEN 2.3, blue edition). Images were taken by two observers in separate laboratories. Continuous organoid monitoring was enabled by image acquisition with 5x magnification at ten times of recording on days 2, 5, 8, 10, 12, 16, 19, 22, 25, and 30 (Fig. 1). Individual organoids were traced by unique identifiers throughout the whole protocol.

### ***In silico*** methods

Organoid size, defined as the number of pixels covered by the organoid, was determined using semantic segmentation which aims to assign each image pixel to one of two classes: organoid or background. For benchmarking, we manually created a reference ground truth of organoid segmentations using^18^. We compared the two classical methods CellProfiler and OrganoSeg, and the machine learning-based methods MOrgAna and SegFormer for automated organoid growth monitoring.

#### Classical methods

- CellProfiler
  CellProfiler is a tool for automated images analysis for a wide range of biological applications^7^. For organoid segmentation, we used the same parametrization as suggested in^9^ for brain organoid segmentation. First, image smoothing was applied using morphological opening and closing operations, employing a structuring element with a diameter of 25 pixels. Subsequently, image intensities were inverted using the ImageMath module. The identification of primary objects was achieved through Global Otsu segmentation, utilizing a two-class thresholding approach. Lastly, to eliminate debris, the analysis focused solely on the largest identified object, by employing the MeasureObjectSizeShape method followed by FilterObjects.
- OrganoSeg

OrganoSeg is an analysis tool for segmentation, filtering, and analysis for organoid brightfield images^8^. As done in^9^ for brain organoid segmentation, we used the default pipeline for segmentation. This corresponds to using Intensity Threshold = 0.5, Window Size = 500, and Size Threshold = 5000. To remove debris, objects smaller than the largest identified object were subsequently excluded.

#### Machine learning-based methods

- MOrgAna-based approach
  The previously published tool MOrgAna provides methods for organoid segmentation, quantification, and visualization of morphological information^9^. It provides a graphical user interface (GUI) for broad application in research. One central MOrgAna module is organoid segmentation which computes pixel-wise features and subsequently classifies those pixels for organoid segmentation. The authors implemented two models: Multilayer Perceptron (MLP) and Logistic Regression (LR). For each model, MOrgAna outputs two masks: ‘classification mask’ (maskC) and ‘watershed mask’ (maskW). To identify the best-performing method, we evaluated both masks for MLP (MOrgAna_MLP,C_ and MOrgAna_MLP,W_) and LR (MOrgAna_LR,C_ and MOrgAna_LR,W_) that were separately trained. For all methods, default parameters were used. The MOrgAna GUI was used for training and inference. During the evaluation, we considered the organoid border, which is separately predicted by MOrgAna, as background.
- SegFormer-based approach

SegFormer is a state-of-the-art transformer-based deep-learning model for semantic segmentation^14^. Python implementations are publicly available online^19^, however require programming and deep-learning experience for model training and inference. For fast training, we used the SegFormer with the smallest implemented encoder (MiT-B0). For model training, evaluation, and inference, the implementation from^19^ was used. We trained the SegFormer with AdamW (learning rate = 0.0001, β1 = 0.9, β2 = 0.999, weight decay = 0.1) using batch size 2 for 1000 iterations. The model used a weighted combination (1:10) of binary cross-entropy and Dice loss. On-the-fly image augmentation included four steps: (1) image downscaling to 256 × 192, which resembles the MOrgAna default downscaling, (2) random flip with a probability of 0.5, (3) z-score normalization, and (4) adding Gaussian Noise (variance range: 0.01-0.1). These augmentations are implemented in^19^ and are commonly used for other semantic segmentation tasks like Cityscapes or ADE20K.

### Model evaluation

**Fig. 2:**
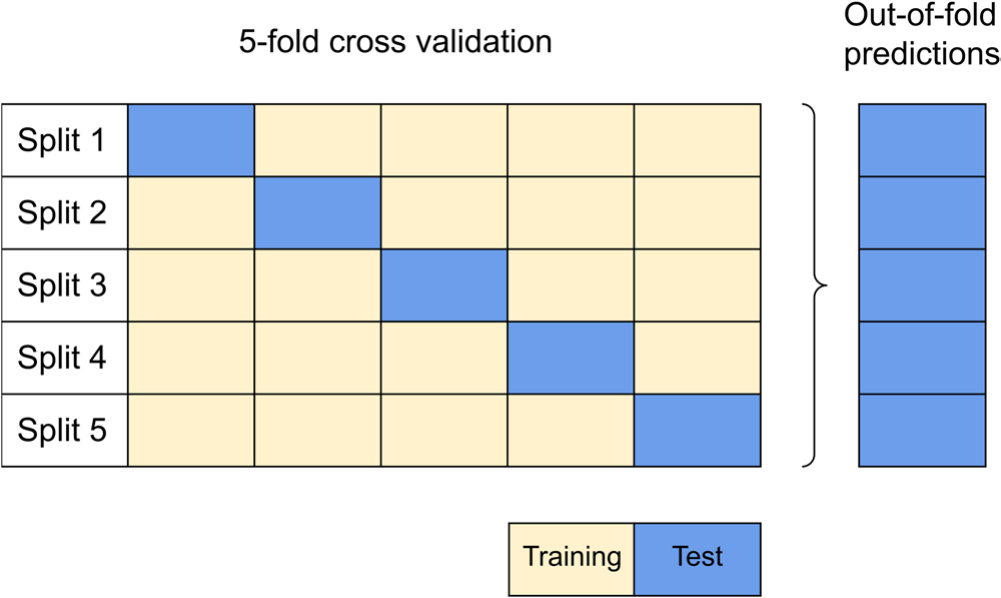
5-fold cross-validation and out-of-fold predictions.

For the evaluation of the machine learning-based methods, we used 5-fold cross-validation (CV). We used it for two reasons: in order to compare the segmentation performance of MOrgAna and SegFormer on multiple data splits, and to derive the model predictions on the complete dataset using the so-called out-of-fold predictions (Fig. 2). Since the models are never exposed to the CV test set during training, out-of-fold predictions are a reliable estimator of segmentation performance. The 5-fold CV splits are (1) based on organoid IDs to ensure that all images of the same biological sample are either in the training or test split and (2) stratified by clone to reduce the model bias towards a specific clone. For the SegFormer, we split the CV training data into an 80% training, 20% validation split for model selection. Since MOrgAna does not allow predefined splits, it generates its own internal training and validation split from the CV training data.

### Model comparison

We used the Dice score for comparing the models’ segmentation performance. The Dice score is a common metric to measure the performance of image semantic segmentation methods. It is defined as two times the area of the intersection divided by the total number of pixels in the ground truth A and predicted segmentation B (Eq. below). A perfect segmentation corresponds to a Dice score of 1.

$$Dice\,score=\frac{2\cdot \left|A\cap B\right|}{\left|A\right|+\left|B\right|}$$

The organoid size in pixels is only a relative measure as it depends on the microscopy magnification and image resolution. To derive the absolute organoid size, we converted the organoid size in px2 to organoid size in μm^2^ (Eq below).

$$org\_siz{e}_{\mu {m}^{2}}=org\_siz{e}_{p{x}^{2}}\cdot {(\mu m\_per\_px)}^{2}$$

$$\mu m\_per\_p{x}_{labA}=\frac{500\,\mu m}{158\,px}$$

$$\mu m\_per\_p{x}_{labB}=\frac{500\,\mu m}{167\,px}$$

### Clone diversity

To quantify the morphology of the different clones, we used PyRadiomics^20^ to extract the following 2D organoid features: Elongation, MajorAxisLength, MaximumDiameter, MeshSurface, MinorAxisLength, Perimeter, PerimeterSurfaceRatio, PixelSurface, and Sphericity. For visualization of clone diversity, we applied z-score normalization to these features and subsequently conducted Principal Component Analysis.

## Data Records

Our dataset is publicly available on Zenodo^21^ at 10.5281/zenodo.10301912. It encompasses two sources of data:

1. A comma-separated values (‘CSV’) file. This file serves as a key to our dataset with one image per row. An excerpt of this file is shown in Table 1. Each image is represented by its image identifier (‘img_id’) with the format [org_id]_[clone]_d[imaging_day]_[imaging_lab]. For each image, the CSV file also specifies the organoid size for convenience. Alternatively, the organoid size can be calculated using the ground truth organoid segmentation (org_seg_GT_).

**Table 1:** Dataset overview.

org_id	img_id	Day	Clone	Imaging	org_size_px2	org_size_µm2
1	org01_wt2D_d02_LabA	2	wt2D	LabA	34246	342954
1	org01_wt2D_d02_LabB	2	wt2D	LabB	37110	332658
⋮	⋮	⋮	⋮	⋮	⋮	⋮
64	org64_B2A-2_d30_LabA	30	B2A-2	LabA	357778	3582939
64	org64_B2A-2_d30_LabB	30	B2A-2	LabB	346590	3106870

**Fig. 3:**
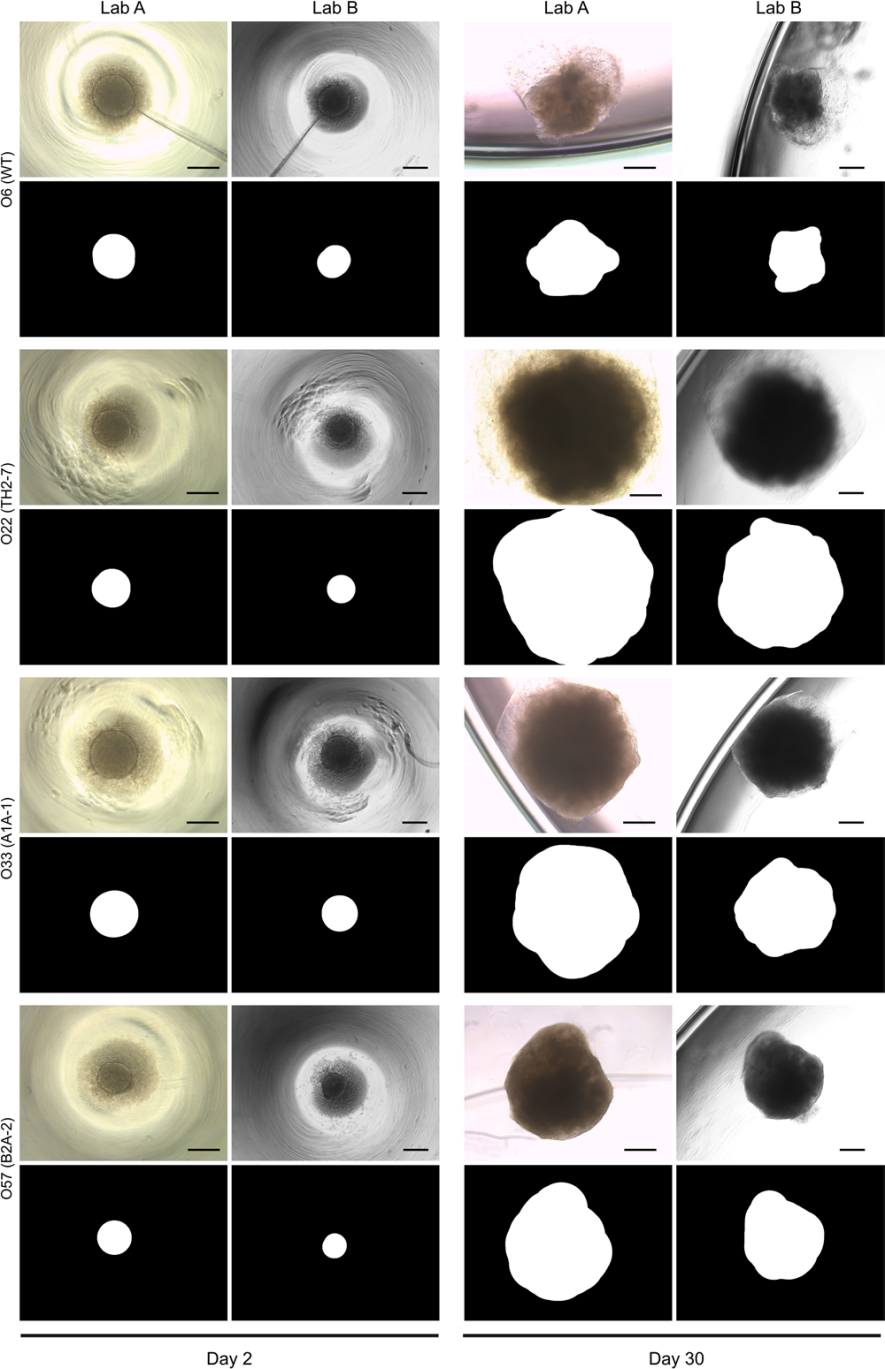
Excerpt of the dataset. One exemplary organoid per clone and the corresponding ground truth organoid segmentation for day 2 and day 30 for imaging in Lab A and Lab B shows heterogeneous organoid development. At day 2, the dark textured region around the centered circular region represents cell debris around the embryoid body. At day 30, the translucent circumferential structures are Matrigel matrix in which the organoids are embedded. Scale bar: 500 μm.

Each image is characterized by (1) the clone the organoid belongs to, (2) the image acquisition day, and (3) the imaging lab. The organoid size in px^2^ and μm^2^ is provided for convenience, however, this can also be calculated from the ground truth organoid segmentations.
2. For each row of the CSV file, we provide the image and org_seg_GT_ (Fig. 3). For Lab A, the images are in JPEG format. For Lab B, the images are in TIF format. Org_seg_GT_ is a manually created binary 2D NumPy array with the same size as the image (1024 × 768 for Lab A, 1388 × 1040 for Lab B). A value of 1 in org_seg_GT_ at position (x, y) means that the same position (x, y) in the corresponding image is covered by the organoid (Fig. 3). The image file and the org_seg_GT_ file have the following format: [img_id].[jpg|tif] and [img_id].npy. For day 12, organoids were imaged before and after embedding from 96-well plates in 12-well plates, allowing the investigation of well-specific optical properties (Supplementary Figure 1). One record of organoid 50 (day 12, Lab A, after embedding) was excluded from the dataset as the image only showed the microscopy background.

## Technical Validation

### Dataset for benchmarking of organoid analysis pipelines

#### Segmentation performance

**Fig. 4:**
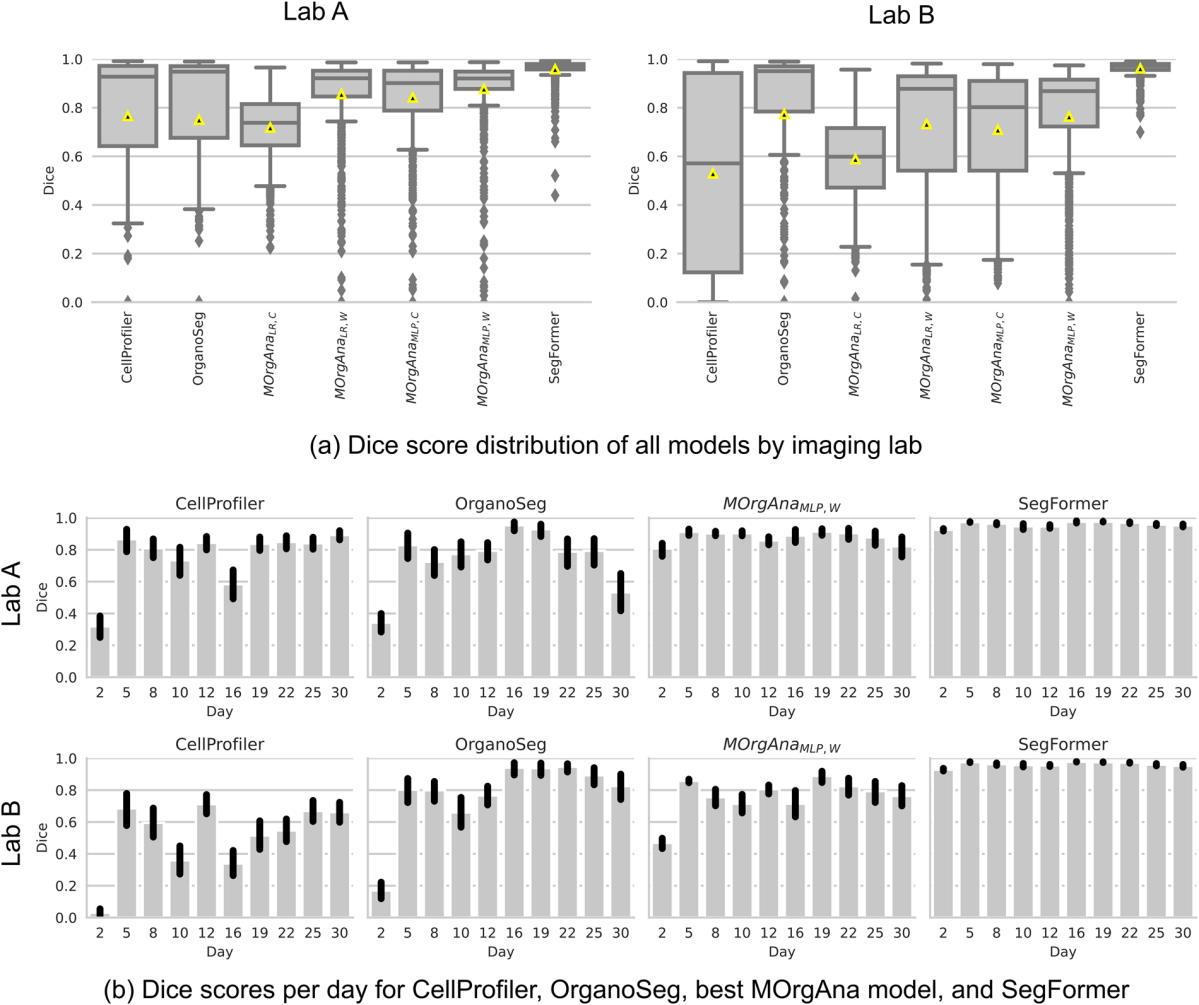
Cross-laboratory images and frequent imaging provide a differentiated view on the segmentation performance of organoid analysis pipelines. The dataset shows a heterogeneous performance of CellProfiler, OrganoSeg, MOrgAna, and the SegFormer for different days and imaging labs. Error bar in (**b**): confidence interval (95%).

As the dataset^21^ contains images of the same organoids from two imaging labs and imaged during the entire course of organoid development, it allows to benchmark the versatility and applicability of different methods for organoid segmentation which is the basis for subsequent organoid growth monitoring. Exemplarily, the two classical methods CellProfiler and OrganoSeg, and the two machine-learning-based methods MOrgAna and the SegFormer were selected to show how the dataset provides a differentiated view on organoid segmentation performance (Fig. 4).

For Lab A, MOrgAna_MLP,W_ is the best MOrgAna configuration reaching a Dice score of 0.88 ± 0.15 (mean ± SD) and outperforms CellProfiler and OrganoSeg which reach Dice scores of 0.77 ± 0.30 (mean ± SD) and 0.75 ± 0.36 (mean ± SD), respectively. The SegFormer outperforms all models, reaching a Dice score of 0.96 ± 0.05 (mean ± SD). For Lab B, OrganoSeg and MOrgAna_MLP,W_ perform similarly reaching Dice scores of 0.77 ± 0.34 (mean ± SD) and 0.76 ± 0.24 (mean ± SD), respectively. The SegFormer outperforms all models here, reaching a Dice score of 0.96 ± 0.03 (mean ± SD). The dataset also reveals limited generalizability of CellProfiler and MOrgAna_MLP,W_ which perform better for Lab A compared to Lab B.

The day-wise organoid imaging demonstrates that the segmentation performance of CellProfiler, OrganoSeg, and MOrgAna_MLP,W_ for Lab B strongly varies from day to day while the SegFormer accurately segments organoids throughout the complete observation time and for both imaging labs (Fig. 4b). Furthermore, the dataset shows that especially CellProfiler and OrganoSeg show a weak performance on images from Day 2 (Fig. 4b).

#### Model robustness

The dataset^21^ contains diverse organoid phenotypes and common imaging distractors which allows to investigate the robustness of organoid analysis pipelines.

**Fig. 5:**
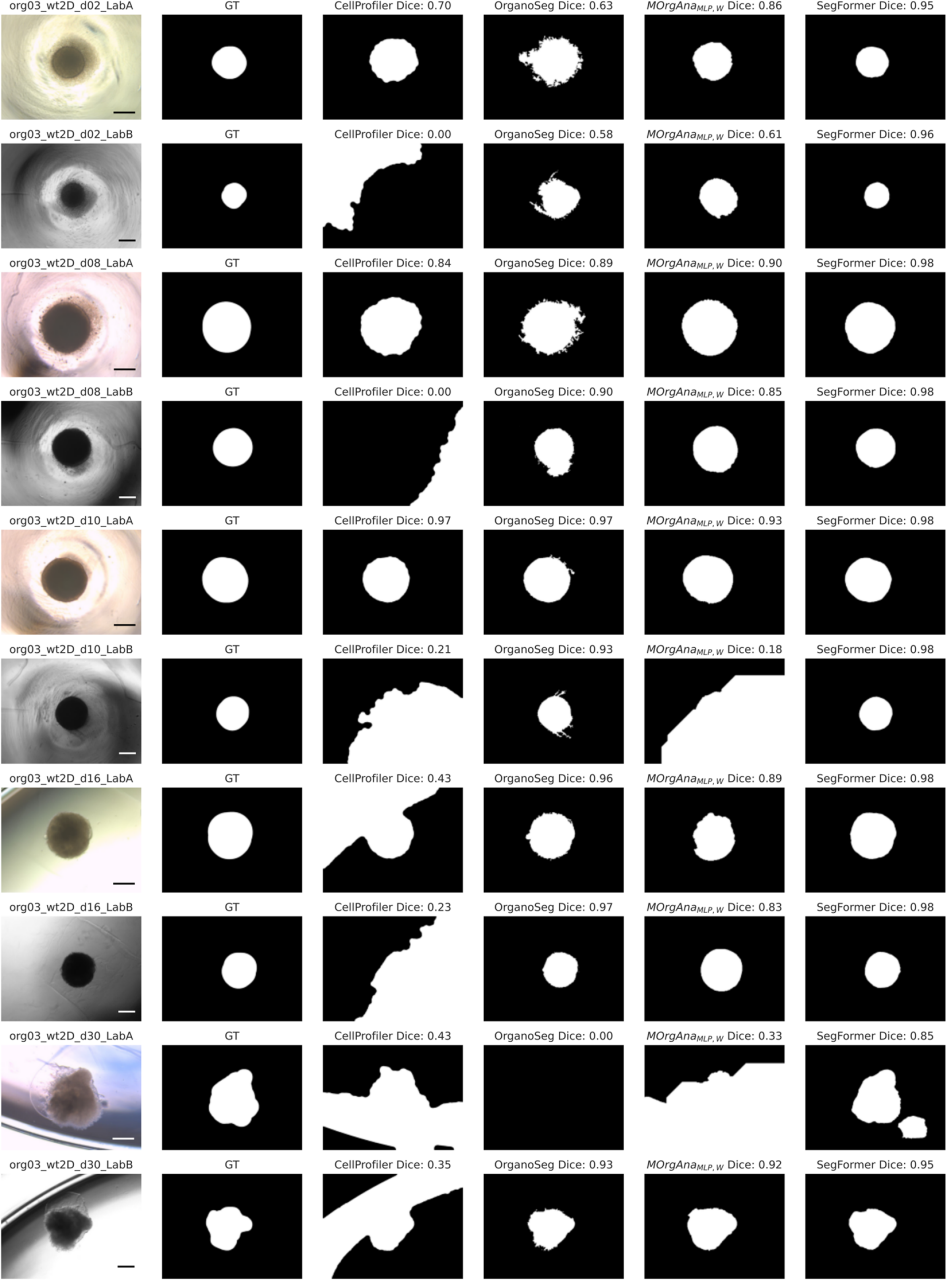
Heterogeneous organoid phenotypes and different imaging features show robustness of organoid analysis pipelines. Shown are images of organoid 3 for days 2, 8, 10, 16, and 30, for both imaging labs in the first column. Columns 2–6 show ground truth (GT) organoid segmentation, CellProfiler prediction, OrganoSeg prediction, MOrgAna_MLP,W_ prediction, and SegFormer prediction. At day 2, the dark textured region around the centered circular region represents cell debris around the embryoid body. At day 30, the translucent circumferential structures are Matrigel matrix in which the organoids are embedded. Scale bar: 500 μm.

First, the dataset shows the robustness of analysis pipelines to day-2 matrigel-surrounded organoids (Fig. 5). For one of those organoids, CellProfiler segments only the background for Lab B. For CellProfiler, OrganoSeg, and MOrgAna_MLP,W_ for both imaging labs, the models erroneously classify the surrounding matrigel as organoid. The SegFormer on the other hand correctly recognizes the organoid border.

Second, it demonstrates the model robustness to the presence of distractors including light reflexes due to rims of plate wells or shadows and different colors caused by culture medium. Occasionally, CellProfiler and MOrgAna_MLP,W_ misclassify dark background as organoid for example for organoids on days 2, 8, 10, 16, 30 (CellProfiler) and days 10 and 30 (MOrgAna_MLP,W_, Fig. 5). For day 30 and Lab A, the SegFormer erroneously segments two organoids instead of one.

#### Organoid growth monitoring

**Fig. 6:**
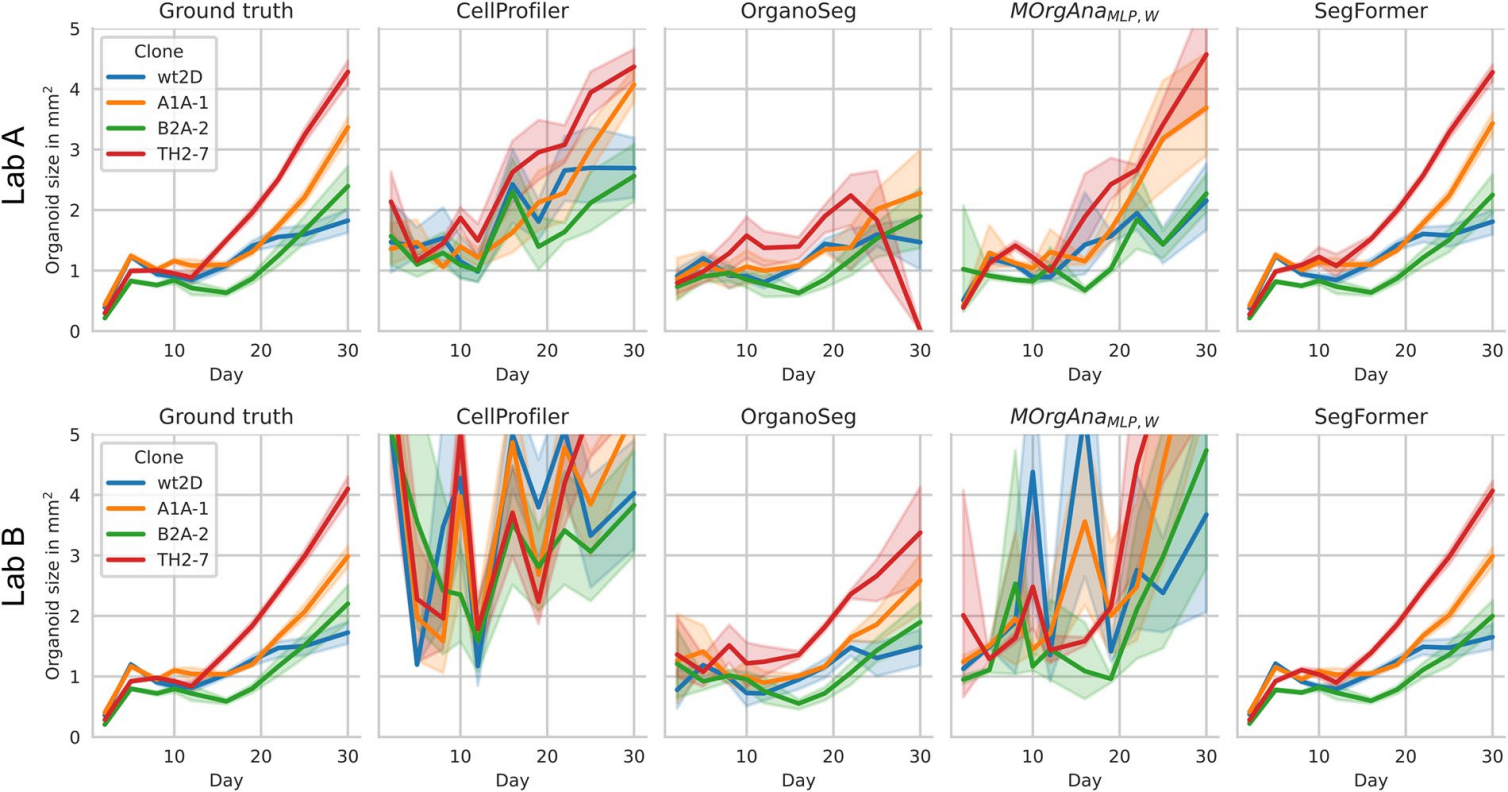
Clones with distinct growing patterns for comparison of organoid analysis pipelines. The rapid decrease in organoid size between days 5 and 8 is due to the transfer of organoids from the V-bottom 96-well to flat-bottom 24-well plates as V-bottom plates optically enlarge the organoids. For MOrgAna_MLP,W_, and SegFormer, all model predictions are from out-of-fold predictions.

The dataset^21^ is ideal for benchmarking organoid growth as the four included clones show clearly distinct growing patterns (Fig. 6). TH2-7 grows fastest, A1A-1 grows second fast. B2A-2 has a growing delay compared to wt2D but catches up until the end of the observation period.

An ideal model for organoid growth monitoring, resembles exactly this pattern. The SegFormer almost perfectly represents the ground truth of organoid growth for both imaging labs (Fig. 6). OrganoSeg for Lab B roughly resembles the actual organoid growth. OrganoSeg for Lab A, CellProfiler, and MOrgAna_MLP,W_ show large deviations from the ground truth organoid growth, thus completely failing with reproducing the actual organoid growth pattern.

Additional to the visual observation of correctly resembling organoid growth, the ground truth organoid annotations allow to calculate the models’ maximum day-wise deviation of the actual organoid size (Supplementary Table 1). This shows that the SegFormer outperforms the remaining models for 8 of 10 days for Lab A and all days for Lab B with ±7% maximum day-wise deviation of the ground truth organoid size. CellProfiler, OrganoSeg, and MOrgAna_MLP,W_ show maximum day-wise deviations of the ground truth organoid size of 1768%, 303%, and 351% (Supplementary Table 1).

### Clone diversity and similarity

**Fig. 7:**
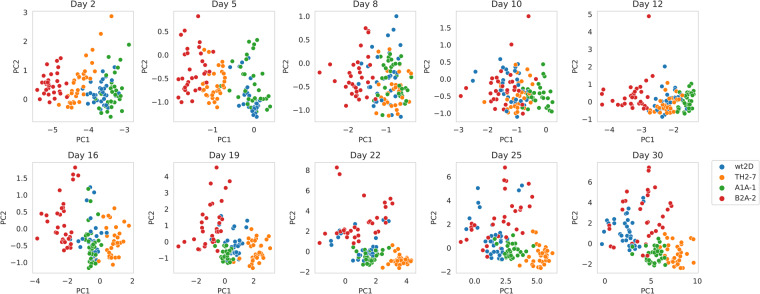
Day-wise diversity of clones. First two principal components of Principal Component Analysis based on nine z-score normalized PyRadiomics 2D imaging features for each imaging day.

Organoid morphology analysis revealed that the clones exhibit different morphologies (Fig. 7). Especially A1A-1, TH2-7, and B2A-2 are rather clearly separated for the majority of days (2, 5, 12, 16, 19, 22, 25). WT2D seems to be similar to A1A-1 on days 2, 5, 8, 16, 19, 22, and 25.

### Supplementary information

Supplementary Information

## Data Availability

The code for training MOrgAna and the SegFormer is publicly available on GitHub: https://github.com/LabTrivedi/MOrgAna and^19^. The data splits for MOrgAna and SegFormer training and evaluation, the configuration files for SegFormer training, the CellProfiler project as well as the workflow for the Technical Validation are publicly available on GitHub and co-deposited on Zenodo^22^.
