# Supplementary material for: A large and diverse brain organoid dataset of 1,400 cross-laboratory images of 64 trackable brain organoids
Source: Sci Data. 2024 May 20;11:514. doi: 10.1038/s41597-024-03330-z (PMC11106320; doi:10.1038/s41597-024-03330-z)
Supplement: Supplementary file 1 — Supplementary Information [file 41597_2024_3330_MOESM1_ESM.docx]

**Supplementary Information**

**Table of contents**

Page 2 – Supplementary Figure 1

Page 3 – Supplementary Table 1 + 2

**
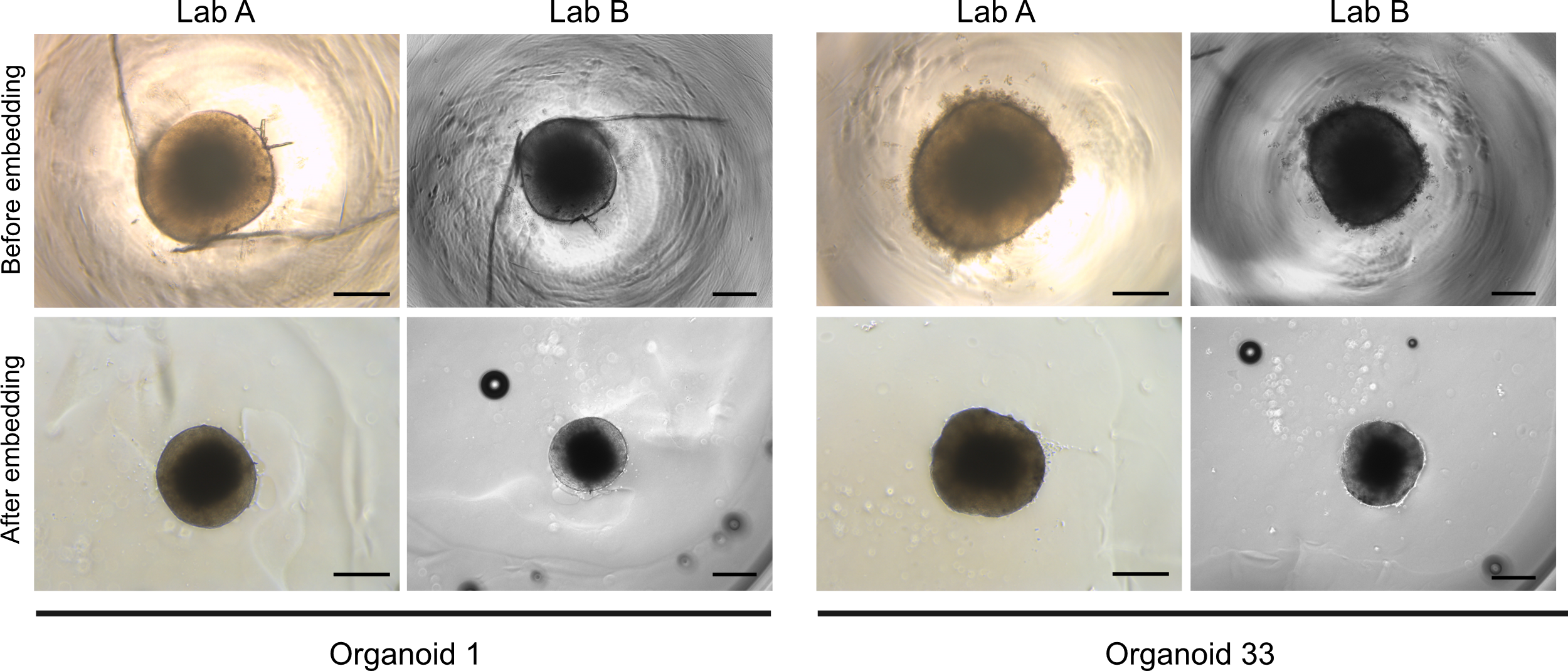
**

**Supplementary Figure 1:** For day 12, the dataset contains images before and after embedding of 96-well plates into 12-well plates to investigate well-specific optical properties. Scale bar: 500 μm.

**Supplementary Table 1: Absolute and relative mean difference from ground truth organoid size.** Best model for each imaging lab and day in bold.

| **Lab A** | | | | | | | | |
| --- | --- | --- | --- | --- | --- | --- | --- | --- |
|  | CellProfiler | | OrganoSeg | | MOrgAna_MLP,W_ | | SegFormer | |
| Day | abs (μm^2^) | rel | abs (μm^2^) | rel | abs (μm^2^) | rel | abs (μm^2^) | rel |
| 2 | +1,297,875 | +436% | +489,611 | +163% | +250,687 | +117% | -12,902 | **-4%** |
| 5 | +207,585 | +21% | -22,707 | -1% | +59,521 | +7% | +222 | **+0%** |
| 8 | +404,867 | +47% | +95,281 | +11% | +188,018 | +20% | +17,124 | **+2%** |
| 10 | +414,076 | +44% | +136,576 | +16% | +29,604 | **+4%** | +60,645 | +7% |
| 12 | +294,134 | +32% | +109,127 | +10% | +182,146 | +30% | +58,271 | **+6%** |
| 16 | +1,164,818 | +133% | -36,974 | -3% | +206,814 | +18% | +13,877 | **+1%** |
| 19 | +685,425 | +52% | -1,897 | **0%** | +287,936 | +21% | +17,595 | +1% |
| 22 | +648,911 | +45% | -220,978 | -13% | +445,152 | +42% | +28,237 | **+2%** |
| 25 | +768,933 | +41% | -433,490 | -15% | +188,689 | +7% | -26,257 | **-2%** |
| 30 | +456,924 | +22% | -1,551,214 | -44% | +205,316 | +11% | -24,459 | **-1%** |
| Best | 0/10 | | 1/10 | | 1/10 | | 8/10 | |
| Max dev. | +1,297,875 | 436% | +489,611 | 163% | +250,687 | 117% | 60,645 | 7% |
| **Lab B** | | | | | | | | |
|  | CellProfiler | | OrganoSeg | | MOrgAna_MLP,W_ | | SegFormer | |
| Day | abs (μm^2^) | rel | abs (μm^2^) | rel | abs (μm^2^) | rel | abs (μm^2^) | rel |
| 2 | +5,218,581 | +1,768% | +839,366 | +303% | +1,019,063 | +351% | +9,667 | **+3%** |
| 5 | +1,229,865 | +142% | +127,355 | +14% | +327,160 | +33% | -5,343 | **-1%** |
| 8 | +1,474,562 | +173% | +238,197 | +28% | +1,119,394 | +137% | +41,078 | **+5%** |
| 10 | +2,989,813 | +334% | +57,620 | +8% | +1,454,328 | +172% | +28,118 | **+4%** |
| 12 | +694,634 | +72% | +52,156 | +6% | +634,079 | +82% | +9,187 | **+1%** |
| 16 | +3,261,957 | +362% | -47,413 | -5% | +1,887,958 | +194% | +4,363 | **+1%** |
| 19 | +1,602,653 | +154% | -62,820 | -6% | +354,102 | +29% | -4,402 | **-1%** |
| 22 | +2,690,624 | +192% | -41,482 | -3% | +1,283,918 | +80% | +770 | **0%** |
| 25 | +1,891,115 | +103% | -202,161 | -11% | +1,925,530 | +101% | -61,049 | **-4%** |
| 30 | +2,075,434 | +84% | -415,567 | -14% | +2,973,921 | +115% | -76,920 | **-3%** |
| Best | 0/10 | | 0/10 | | 0/10 | | 10/10 | |
| Max dev. | +5,218,581 | 1,768% | +839,366 | 303% | +1,019,063 | 351% | +41,078 | 5% |

**Supplementary Table 2: Comparison of training time for one cross-validation split.** Run on a machine with one NVIDIA GeForce RTX 3090 graphics card. Training times for the MOrgAna models are independent of whether classification or watershed masks are used as those are only generated during inference.

| **Model** | **Training time (s)** |
| --- | --- |
| MOrgAna_LR_ | 248 |
| MOrgAna_MLP_ | 1196 |
| SegFormer | 658 |
